# Characterization of the Key Aroma Compounds in Traditional Hunan Smoke-Cured Pork Leg (Larou, THSL) by Aroma Extract Dilution Analysis (AEDA), Odor Activity Value (OAV), and Sensory Evaluation Experiments

**DOI:** 10.3390/foods9040413

**Published:** 2020-04-02

**Authors:** Dandan Pu, Yuyu Zhang, Huiying Zhang, Baoguo Sun, Fazheng Ren, Haitao Chen, Yizhuang Tang

**Affiliations:** 1 Beijing Key Laboratory of Flavor Chemistry, Beijing Technology and Business University (BTBU), Beijing 100048, China; 18518351472@163.com (D.P.); zhanghuiying@btbu.edu.cn (H.Z.); sunbg@btbu.edu.cn (B.S.); chenht@th.btbu.edu.cn (H.C.); yizhuang.tang@foxmail.com (Y.T.); 2 Beijing Advanced Innovation Center for Food Nutrition and Human Health, College of Food Science & Nutritional Engineering, China Agricultural University, Beijing 100083, China; renfazheng@cau.edu.cn

**Keywords:** traditional Hunan smoke-cured pork leg, gas chromatography–olfactometry, aroma compounds, recombination and omission test

## Abstract

The key aroma compounds in smoke-cured pork leg were characterized by gas chromatography–olfactometry coupled with aroma extract dilution analysis (GC–O/AEDA), odor activity value (OAV), recombination modeling, and omission tests. Ranking analysis showed that pork leg smoke-cured for 18 days had the best sensory qualities, with strong meaty, smoky, roasty, woody, and greasy attributes. Thirty-nine aroma-active regions with flavor dilution (FD) factors ranging from 9 to 6561 were detected. Overall, 3-ethylphenol had the highest FD factor of 6561, followed by 2,6-dimethoxyphenol, 3,4-dimethylphenol, 4-ethylguaiacol, 4-methylguaiacol, 3-methylphenol, and 2-acetyl-1-pyrroline, with FD ≥243. Among 39 aroma compounds, 27 compounds with OAVs ≥1 and were potent odorants. A similarity of 90.73% between the recombination model and traditional Hunan Smoke-cured Pork Leg (THSL) sample was obtained. Omission tests further confirmed that (*E*)-2-nonenal, 2-methoxy-4-vinylphenol, guaiacol, 3-ethylphenol, 2,6-dimethylphenol, 2-acetyl-1-pyrroline, and methional were key odorants in smoke-cured pork leg. Additionally, 2-acetyl-1-pyrroline (38.88 μg/kg), which contributes to a roasty aroma, was characterized here as a key odorant of smoke-cured pork leg for the first time.

## 1. Introduction

Pork is an important source of food that provides rich nutrients, such as protein and fat. The production of pork reached 5415 tons globally in 2018, and its consumption in China reached 40 kg/capita/year (data from the China Industry Information Network, May 2019). Compared to fresh pork, smoke-cured pork has a richer aroma, a smoky and savory aroma that is considered to be unique. Smoke-cured processing extends the shelf-life and enhances the flavor quality of meat products [1]. Hunan smoke-cured meat, with its bright red color and strong smoky and savory aroma, is one of the most famous smoke-cured meats in China. There are two kinds of Hunan traditional smoke-cured meat products: smoke-cured bacon and smoke-cured pork leg (THSL). The processing procedures for THSL include meat cutting and trimming, salt-curing, hang-drying, smoke-curing, and packaging. Smoke-curing is the main process that plays an important role in the generation of the meat’s unique smoky and savory aroma. Aroma is the key factor that decides the sensory quality of the food and its appeal to consumers [2]. During the production of smoke-cured meat, many aroma compounds are generated through microbial fermentation [3,4], lipid oxidation [5,6,7], the Maillard reaction, and Stecker degradation [3]. These aroma compounds contribute to the aroma profiles of smoke-cured meat products.

More than 400 volatile compounds in smoke-cured meat products have been reported in previous studies. It is widely known that not all volatile compounds in foods contribute to their aromas. Only a small number (~1–3%) of such compounds can be detected as key odorants by human olfactory receptors, which results in aroma perception [8]. By applying gas chromatography–olfactometry (GC–O) coupled with the frequency method, 3-ethylphenol, guaiacol, furfuryl alcohol, 2,5-dimethlpyrazine, (*E*,*E*)-2,4-decadienal, and methional were identified as key odorants in smoke-cured mini-pig [6]. In addition, 2-methyl-3-(methyldithio)furan, 2-methyl-3-furanthiol, guaiacol, and 1-octen-3-one were characterized as the most potent odorants in smoked cooked loin by GC–O coupled with aroma extraction dilution analysis (AEDA) [9]. In our previous study, 2-methylpyrazine, 3-methyl-2-cyclopenten-1-one, nonanal, 5-methyl furfural, benzaldehyde, guaiacol, and eugenol were characterized by GC–O as aroma-active compounds in industrially produced Hunan smoke-cured bacon [10]. Using solid-phase microextraction (SPME), Yu and co-workers identified 27 and 43 volatile compounds, including four phenolic compounds in Hubei smoke-cured bacon with different fiber coatings [11,12]. Moreover, the regularity of changes in aroma during the smoke-curing process for Hunan smoke-cured meat and Chongqing smoke-cured meat was analyzed by SPME coupled with gas chromatography–mass spectrometry (GC–MS) analysis and simultaneous distillation extraction (SDE) coupled with GC–MS analysis, respectively [13,14]. During the smoke-curing process, there is a significant increase in the concentrations of phenolic and carbonyl volatiles—the major aroma compounds in Hunan smoke-cured bacon [14,15]. Saldaña and co-workers reported that “woody” and “pleasant” aroma attributes correlated with a high number of related volatile compounds, mainly aldehydes, phenols, and ketones [16]. Moreover, some volatile compounds, such as (*E,E*)-3-octen-2-one, 2-octenal and (*E*)-2-tridecenal, are responsible for the rancid off-flavor of smoke-cured meat during storage [17]. Although many related studies have been conducted, most of them have focused on the composition and the identification of volatile constituents and how these generally change during smoke-curing processing including the effects of different smoke-cured woods on the volatile compounds and their aroma profile properties [18,19]. Many of the key aroma compounds in smoke-cured meat products remain to be identified. Key odorants are the main reference compounds used to clarify the quality differences between different brands of food and are also the marker compounds used to monitor quality during food production. Moreover, the characterization of key aroma compounds could provide a way to modify or change the manufacturing process of smoke-cured pork leg [20].

Therefore, the objective of this work was to identify the key aroma compounds in THSL by using a systematic approach including the (1) isolation of volatile compounds by simultaneous distillation extraction (SDE); (2) identification of aroma-active compounds by GC–O–MS/AEDA; (3) quantification of aroma-active compounds through construction of standard curves and calculation of the odor activity values (OAVs); and (4) validation and confirmation of key odorants by recombination modeling and omission experiments.

## 2. Materials and Methods

### 2.1. Sample Preparation

**Figure 1:**
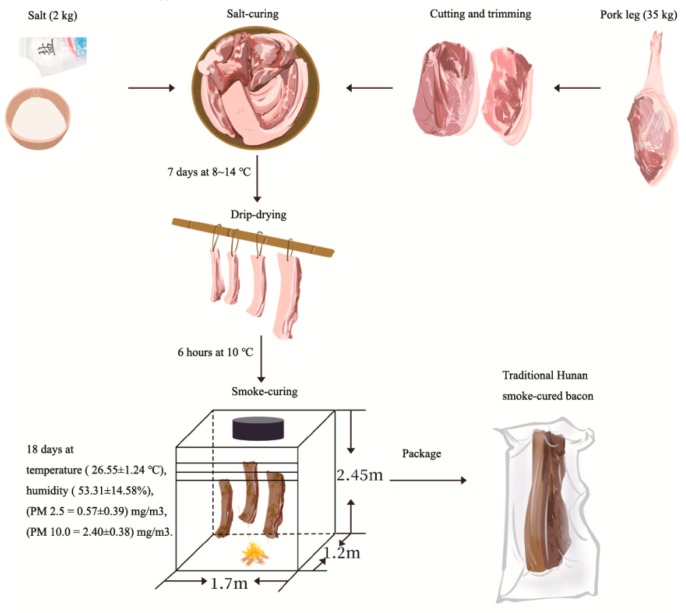
Steps for the preparation of traditional Hunan smoke-cured pork leg (THSL).

The THSL was produced in Huaihua, Hunan province, China. The specific preparation steps for the THSL sample are shown in Figure 1. The pork leg (*Duroc* pig, Hunan white pigs line 1) was purchased from a local farmer (Huaihua, China). The pork leg was cut into pieces (~4–5 cm in width, ~40–50 cm in length) and trimmed. These pieces were then salt-cured (using 35:2 mass ratio of meat to salt) for 7 days. After 6 h of hang-drying to drain out the blood and water, the salt-cured sample was smoke-cured by burning the stem portion of dried *Camellia oleifera* Abel sticks (~50–55 cm) at a temperature of 26.55 ± 1.24 °C and humidity of 53.31% ± 14.58% for 30 d. The concentration of smoke (PM 2.5 = 0.57 ± 0.39 mg/m^3^, PM 10.0 = 2.40 ± 0.38 mg/m^3^) was monitored using a PC-3A Dust Instrument (Qingdao, YiLan Environment, China) during the smoke-curing process. During the smoke-curing process, the THSL sample was sampled daily (coded THSL-1, THSL-2, …, and THSL-30) using triplicates for each sample.

### 2.2. Chemicals

Dichloromethane and 1,2-dichlorobenzene (HPLC grade) were purchased from Thermo Fisher (Beijing, China). 2-Ethylnaphthalene, 2-methylnaphthalene, 3-ethyl-2-hydroxy-2-cyclopenten-1-one, 1-methylnaphthalene, 2,3,5-trimethylpyrazine, octanal, guaiacol, 5-methyl furfural, 3-methyl-2-cyclopenten-1-one, 2-methylpyrazine, 2-methylphenol, 3-methylphenol, 2-acetyl-1-pyrroline, and methional with a purity over 99% were purchased from J&K (Beijing, China). 3-Methylacetophenone, (*E*)-2-octenal, 1-octen-3-one, 3,4,5-trimethoxytoluene, 3,4-dimethyltoluene, 2-methoxy-4-vinylphenol, 2-acetylfuran, 3,4-dimethyphenol, 2-methoxy-4-propyl-phenol, and (*E*)-2-nonenal of 97% purity were purchased from Alfa (Beijing, China). 2,6-Dimethoxyphenol, 2,6-dimethylphenol, 3,5-dimethoxyphenol, 2,3-dimethoxyphenol, 2,5-dimethylphenol, and 4-ethylguaiacol with purity over 95% were purchased from Acros (Beijing, China). 4-Methylguaiacol and 3-ethylphenol with 99% purity were purchased from Ark (Beijing, China). Food grade edible salt was purchased from the China Salt Industry Corporation (Beijing, China), and the dried stem portion of *Camellia oleifera* Abel was purchased from a local supermarket (Huaihua, China). Capric triglyceride was purchased from Nanjing Kangmanlin Chemical Industry Co., Ltd. (Nanjing, China).

### 2.3. Sensory Evaluation Analysis

A ranking analysis and quantitative descriptive analysis methods were used in this study. The sensory evaluation was performed by 15 experienced panelists (6 males and 9 females aged 23–28, healthy, without rhinitis, and nonsmokers) recruited from our laboratory. Before evaluation of the THSL sample, the panelists were trained to distinguish and describe the aroma characteristics of 32 pure standards (Section 2.2). Each of the standards was dissolved in the odorless ultra-pure capric triglyceride solution at a concentration of 10 mg/L. After 3 weeks of training (sessions held once per week), the panelists proceeded to conduct sensory evaluation of the THSL sample. Firstly, the ranking analysis was conducted by asking the panelists to sort the THSL samples according to their preferences. The 30 smoke-cured THSL samples were divided into 5 groups (THSL-1 to THSL-6; THSL-7 to THSL-12; THSL-13 to THSL-18; THSL-19 to THSL-24; and THSL-25 to THSL-30). The panelists were asked to select the 2 best samples from each group. Finally, the panelists’ favorite THSL sample was selected from the 10 THSL samples according to selection frequency.

The THSL sample with the best sensory quality (5 g) was cut into pieces (~1–2 mm) and loaded into an odorless transparent plastic bottle (30 mL) which was then presented to the panelists. A unique and random four-digit number written on paper was pasted onto the sample bottle for identification. The panelists were asked to analyze the THSL sample using the 7 sensory attribute profiles (woody, smoky, meaty, milky, greasy, roasty, and spicy) as determined by the sensory evaluation group. The sensory descriptors were evaluated in comparison to capric triglyceride solutions of the corresponding reference odorants: meaty (2-methyl-3-furanthiol, 10 mg/L), greasy ((*E,E*)-2,4-decadienal, 20 mg/L), milky (2,3-butanedione, 10 mg/L), woody (guaiacol, 50 mg/L), smoky (2,6-dimethylphenol, 20 mg/L), roasty (2-acetyl-1-pyrroline, 10 mg/L), and spicy (4-guaiacol, 50 mg/L). Each of the attributes was evaluated on a nine-point scale (1–3, weak; 4–6, medium; 7–9, strong) according to the reference standards. All experiments were repeated three times.

### 2.4. Separation of Volatile Compounds

Following evaluation of the 30 THSL samples, the volatile compounds were isolated using only the THSL sample considered to have the best sensory quality. An improved version of the Likens–Nickerson-type simultaneous distillation extraction (SDE) apparatus was used to extract the volatile compounds from the selected THSL [10,21]. SDE method was chosen because the procedures in this method are similar to that for preparing stewed THSL, a special dish of Hunan. For SDE, a 100 ± 0.2 g THSL sample was cut into pieces (~1–2 mm), mixed with 700 mL distilled water, and then loaded into a 1 L round-bottom flask located in the light phase section of the apparatus, and 50 μL of 1,2-dichlorobenzene dichloromethane solution (1.35 mg/mL) was added. Redistilled dichloromethane (50 mL) was loaded into a 100 mL round-bottom flask in the dense phase section. The water phase was heated in a thermostatic oil bath (Yu Hua, GongYi, China) at 130 °C, and the solvent phase was heated in a thermostatic water bath at 50 °C to speed up the reflux and extraction of the water and solvent phases. Each sample was extracted for 4 h according to procedures described in our previous work [10]. The THSL extract was dried with anhydrous sodium sulfate and then concentrated to 1 mL with a Vigreux column. All experiments were repeated thrice.

### 2.5. GC–MS and GC–O–MS Analysis

The identification and quantification of the aroma compounds were conducted by using a Thermo Fisher Trace 1310 gas chromatograph coupled with a single quadrupole (ISQ) mass spectrometer (GC–MS) (Thermo Fisher, Waltham, MA, USA) in split ratios of 20:1. The GC–MS was equipped with a sniffing port (ODP3, Gerstel, Germany) (GC–O–MS) and the GC effluent was split 1:1 between the MS and sniffing port for the special structure of the GC–O–MS in splitless injection. The separation of the aroma compounds in the THSL extract was achieved on TG-5MS and TG-WAX columns (both 30 m × 0.25 mm i.d. × 0.25 μm film thicknesses, Thermo Fisher). Helium (99.999%) was used as carrier gas at 1.200 and 1.600 mL/min constant flow for GC–MS and GC–O–MS, respectively. The GC–MS and GC–O–MS analysis methods referenced in our previous work were used with some modifications [10,22].

The oven temperature of the TG-WAX column analysis was initially held at 40 °C for 3 min and was then increased to 100 °C at 8 °C/min, to 135 °C at 6 °C/min, held for 3 min, and then increased again to 160 °C at 1.5 °C/min and held for 3 min before being finally increased to 220 °C at 3.6 °C/min, which was held for 4 min. The oven temperature of the TG-5MS column analysis was initially held at 50 °C for 1 min and was then increased to 150 °C at 2.5 °C/min and held for 2 min; finally, it was increased to 220 °C at 10 °C/min and held for 3 min. The temperature of the sniffing port, injector, and ion source were kept at 230, 250, and 280 °C, respectively. Electronic-impact mass spectra ionization mode was used with an ionization energy of 70 eV at full scan mode (*m/z* ranging from 40 to 450 amu).

### 2.6. Aroma Extraction Dilution Analysis (AEDA)

AEDA was used to characterize the flavor dilution (FD) factors of aroma-active compounds. The concentrated organic extract was diluted with dichloromethane solvent in a three-fold dilution series corresponding to 1:3, 1:9, …, 1:X dilutions. An aliquot of each dilution (1 μL) was submitted to GC–O–MS with a TG-WAX column under the same GC conditions described in Section 2.5. All dilutions were repeated in triplicate by three panelists, and data were only recorded for aroma compounds that were detected in at least two replicates. The panelists underwent GC–O–MS training by sniffing 32 standards aroma compounds in a dichloromethane solvent (~50–500 μg/kg) five times before this experiment.

### 2.7. Identification and Quantification

Identification of the aroma compounds was based on a comparison with the mass spectra (MS) database NIST 2014, retention indexes (RIs, on nonpolar and polar GC columns), pure standards (S), and odor characteristics (O). Aroma-active compounds with FD ≥9 were quantified using the standard curves with 50 μL 1,2-dichlorobenzene (1.35 mg/mL) as internal standard. The correction coefficients of each aroma compound were calculated according to 1,2-dichlorobenzene internal standard. The detailed quantification procedures were performed according to our previous study [23,24].

### 2.8. Calculation of the Odor Activity Value

The OAVs of aroma-active compounds were calculated by dividing the ratio of their concentration as measured in the THSL sample to their odor threshold as detected in water. These threshold values were referenced from the literature [25]. The unknown threshold values of the identified aroma-active compounds were detected by a triangular test according to the research measurement method [26]. Two odorless glass vessels (40 mL) filled with 20 mL odorless deionized water and another filled with an odorant standard solution were presented to 15 trained assessors who were asked to identify the differences among the 3 samples by a forced-choice test.

### 2.9. Aroma Recombination

Odorants with OAV ≥1 were dissolved in odorless capric triglyceride at concentration levels equal to those determined in the THSL sample. The recombination model and original THSL sample were evaluated using sensory tests according to the process in Section 2.3.

### 2.10. Omission Tests

To further validate the contribution of certain odorants to the overall aroma profile of the THSL, we prepared a series of omission models according to their high FD factors and high OAVs. Three samples comprising the omitted model and two complete recombination models, all encoded with 4 digital numbers, were presented to the panelists. The omitted model and complete reconstituted model were evaluated by quantitative descriptive analysis according to the process in Section 2.3 and triangle tests.

### 2.11. Statistical Analysis

The sensory evaluation results for each aroma profile were analyzed using one-way analysis of variance (ANOVA) by SPSS 20.0 (SPSS Inc., Chicago, IL, USA). The correlation analysis of the recombination model and the THSL sample was conducted with Microsoft Excel 2016. The confidence interval of the ANOVA analysis, correlation analysis, and standard curve calculation was 95%.

## 3. Results and Discussion

### 3.1. Sensory Evaluation

**Figure 2:**
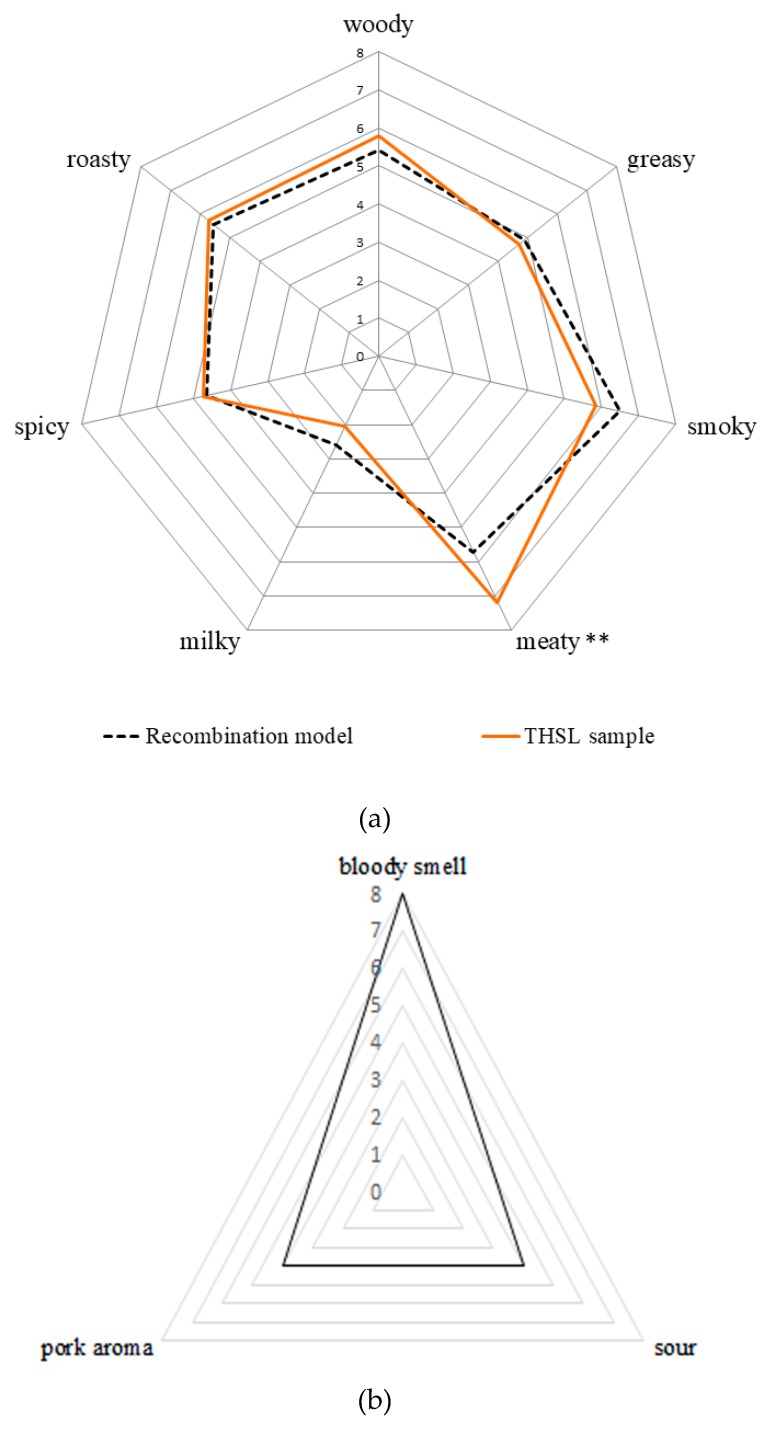
Quantitative descriptive analysis results of the traditional Hunan smoke-cured pork leg (THSL), along with its aroma recombination model (**a**) and the raw material of the pork leg (**b**). **, the significance at *p* < 0.01.

As the ranking analysis results show (Table 1), 10 THSL samples were selected from five groups, and the smoke-cured-18-day THSL (THSL-18) was evaluated to have the best sensory quality based on its high preference frequency of 14. Thus, THSL-18 was selected as the raw material for further study (the QDA analysis and aroma identification experiments). The overall aroma profiles between the raw pork leg and THSL were also compared (Figure 2). A sour smell, pork aroma, and a strong bloody smell constituted the overall aroma of the raw pork (Figure 2a). However, after salt-curing and smoke-curing, the THSL had more complex and diverse aroma profiles, including woody, greasy, smoky, meaty, milky, spicy, and roasty (Figure 2b). The change in the aroma profile going from raw pork to THSL highlights that the salt-curing and smoke-curing processes increased the flavor of the raw pork. As previous studies have shown, many physical and biochemical reactions occur during smoke-cured meat product processing, including microbial fermentation [3], the Maillard reaction [4], lipid oxidation [5,7], Stecker degradation [4,6], and smoke-curing [7,26], leading to the generation of many aroma compounds. Therefore, the bloody and sour aroma attributes of the fresh pork were changed to woody, greasy, smoky, meaty, milky, spicy, and roasty attributes. These results show that food processing techniques can remove off-flavors and promote the aromatic quality of raw meat.

Figure 2 shows that the dominant aroma attributes of THSL were meaty, smoky, roasty, woody, and greasy notes, which is significantly different from the dominant aroma profile of Guangdong bacon (with strong alcoholic, sweet, and sauce-like attributes) [27]. Smoke-cured bacon showed a lower intensity in its meaty, greasy, and roasty notes than those of the THSL [27]. Similar to the Jinhua ham, THSL also exhibited strong meaty and greasy notes. However, the other dominant aroma profiles of the ham included rancid flavor as well as cured, sour, and caramel flavors, which were different from the smoky, woody, roasty, and spicy characteristics of the THSL [28].

### 3.2. GC–O/AEDA Analysis

**Figure 3:**
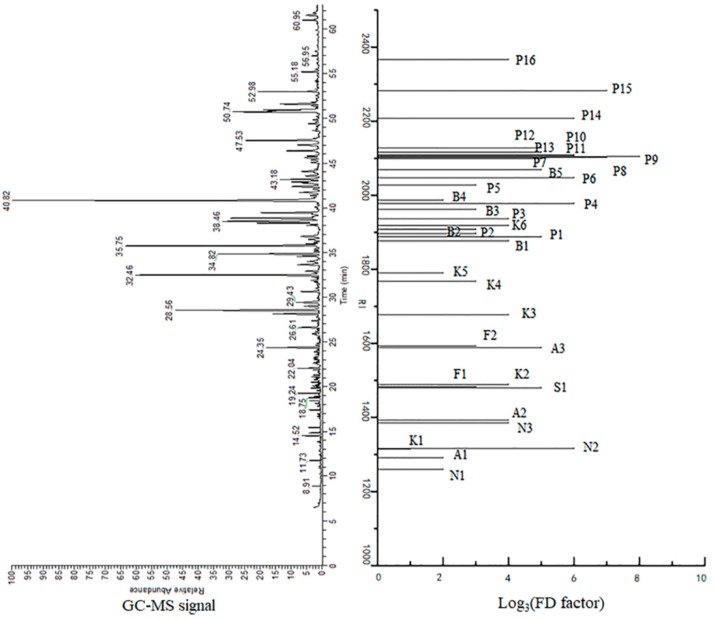
Aromagram and chromatogram of the volatiles isolated from traditional Hunan smoke-cured pork leg (THSL) with their flavor dilution (FD) factors and retention indexes (RIs) on a TG-WAX column according to Table 2.

Through application of the GC–O/AEDA method, 39 aroma-active compounds (including 3 unknowns) with FD factors ranging from 9 to 6561, were identified in the THSL (Table 2). The chromatograms of the aroma compounds isolated from THSL on a TG-WAX column and the aroma-active compounds identified by GC–O/AEDA are shown in Figure 3. The major aroma-active compounds in the THSL were divided into two RI regions: 1200–1600 and 1900–2400 (Figure 3). In addition, most of the aroma-active compounds with high RIs exhibited higher FD factors. Among them, 3-ethylphenol (FD = 6561; leather and smoky) had the highest FD factor, which was consistent with a previous study that detected high aroma frequency and strong intensity in smoke-cured mini-pig [6]. However, 3-ethylphenol was determined to have a low FD factor of 32 in smoked cooked loin [9]. This might have resulted from the use of different smoke-curing techniques, given the smoked cooked loin was only smoke-cured for 20 min, while the THSL was smoke-cured for 18 days and the smoke-cured mini-pig was smoke-cured for 1 month. Additionally, 3,4-dimethylphenol (FD = 2187; stink and leather) and 2,6-dimethoxyphenol (FD = 2187; leather and green) had the second-highest FD factors. It was reported that 2,6-dimethoxyphenol was also detected to have a high FD factor (128) in smoke-cured loin [9]. The 4-methylguaiacol (FD = 729; sweet, woody, caramel-like, and smoky), 4-ethylguaiacol (FD = 729; sweet, woody, caramel-like, and smoky), 2-methoxy-4-propyl-phenol (FD = 729; green, cool, fresh), 3-methylphenol (FD = 729; burning, leather, and stinky), and 2-methoxy-4-vinylphenol (FD = 729; vanilla-like and smoky) ranked third in terms of their FD factors, followed by guaiacol (FD = 243; woody, sweet, and caramel-like), 2-acetyl-1-pyrroline (FD = 243; rice-like and cooked), 3,4,5-trimethoxytoluene (FD = 243; bitter, earthy, and pungent), 2,5-dimethylphenol (FD = 243; stinky and leathery), 2,3-dimethoxyphenol (FD = 243; rubber), and 3,5-dimethoxyphenol (FD = 243; rubber). Most of these aroma-active compounds with high FD factors were phenolic compounds.

The phenolic compounds (P1–P16) with a high intensity of woody, pungent, and smoky characteristics were generated from lignin and phenolic acid degradation during wood-burning or enzymatic degradation by microorganisms [26]. Compared to previous research, these compounds that were detected in THSL were also found in other smoke-cured foods, such as smoked cooked loin [9], Hubei traditional smoke-cured bacon [11,12], Chongqing smoked-cured bacon [15], and traditional smoke-cured bacon of mini-pig [6]. These results are consistent with the previous identification of 3-ethylphenol as a key odorant in traditional smoke-cured bacon of mini-pig [6]. Guaiacol, 4-ethylguaiacol, and 4-methylguaiacol are important aroma-active compounds contributing to the smoky aroma of smoke-cured frankfurters and mini-salami [26]. These results indicate that phenolic compounds play a key role in the overall aroma profiles of THSL.

By contrast, 2-acetylfuran (FD = 27; sweet and roast), octanal (FD = 9; green and citrus), 2-methylpyrazine (FD = 9; green and citrus), 3-methylacetophenone (FD = 9; green and medicine), and 3,4-dimethoxytoluene (FD = 9; green and dried grass) all had relatively lower FD factors. Six ketones (K1–K6) were detected in THSL, five of which had FD ≥9. Among these ketones, 3-methyl-2-cyclopenten-1-one, 3-ethyl-2-cyclopenten-1-one, and 3-ethyl-2-hydroxy-2-cyclopenten-1-one had the highest FD factors (81). Notably, 1-octen-3-one (FD = 3; mushroom) was determined to have the lowest FD factor of all characterized compounds in the THSL. As a previous study reported, many cyclopenten-1-one derivatives were generated during the high temperature degradation of wheat straw hemicellulose [29]. Therefore, 3-methyl-2-cyclopenten-1-one, 3-ethyl-2-cyclopenten-1-one, and 3-ethyl-2-hydroxy-2-cyclopenten-1-one, which present caramel-like and sweet characteristics in THSL, could be derived from wood-burning during the smoke-curing process. As previously reported, most of the methyl ketones, such as 3-hydroxy-2-butanone, 2,3-butanedione, and 2-heptanone in cooked meat, could be derived from lipid degradation [30].

Three nitrogenous compounds (N1–N3) were detected in THSL. 2-Acetyl-1-pyrroline, with an FD factor of 729, was detected here in THSL for the first time. An important aroma compound with roasty and cooked-rice characteristics, 2-acetyl-1-pyrroline can be derived via the Maillard reaction that occurs during fermentation or through smoke-curing (heating). It has been also identified as a critical odorant in rice [31,32], cooked lion [9,16], and bread [33]. Additionally, 2,3,5-trimethylpyrazine (81) and 2-methylpyrazine (9) were also detected in THSL. As previous studies have shown, pyrazines are the main byproduct of the Maillard reaction during the smoke-curing process [4,34]. Three aldehydes (A1–A3) were detected in THSL. (*E*)-2-Nonenal exhibited the highest FD factor with 243, followed by (*E*)-2-octenal (FD = 81) and octanal (FD = 9). Aliphatic aldehydes are mainly generated from the oxidation of unsaturated fatty acids in meat [34,35]. Aldehydes are important odorants contributing to the overall aroma profiles of THSL because of their low threshold values. Five aromatic compounds (B1–B5) were detected in THSL. 3,4,5-Trimethoxytoluene was found to have the highest FD factor of 243. These compounds were mainly derived from wood-burning during the smoke-curing process. Two furan compounds (F1–F2) were detected in THSL, including 2-acetylfuran and 5-methyl furfural with an FD factor of 27. Furan compounds were mainly generated from the Maillard reaction during the smoke-curing process and were also identified as potent odorants in meat products [3,4]. One sulfur compound (S1), methional, was detected in THSL with an FD factor of 243. Methional is produced from methionine with a high intensity of cooked potato characteristics with roasty attributes; it has been identified as a key odorant in smoke-cured mini-pig [6,9].

### 3.3. Quantitation and Calculation of the OAV

Thirty aroma-active compounds (FD ≥ 9) were quantified in THSL. Their quantitative ions and standard curves are shown in Table S1. All calibration curves have good linearity because all correlation coefficients (*R*^2^) were over 0.99. The concentrations and OAV results are presented in Table 3. Phenolic compounds exhibited the highest concentrations among all the compounds, including 2,6-dimethoxyphenol (9784.39 μg/kg), 3,5-dimethoxyphenol (4368.41 μg/kg), 2-methoxy-4-vinylphenol (2761.99 μg/kg), 2,6-dimethylphenol (2455.15 μg/kg), and 2-methylphenol (2316.82 μg/kg). Most of the phenolic compounds with high FD factors exhibited significantly higher concentrations (597.00 to 12,710.00 μg/kg) than other aroma compounds, indicating that they contribute to the overall aroma profiles of THSL [2,9]. However, the amounts of guaiacol, 4-ethylguaiacol, 3-ethylphenol, and 3-methylphenol in THSL were lower than those of smoked cooked loin (guaiacol, 1271.00 μg/kg; 4-ethylguaiacol, 270.00 μg/kg; 3-ethylphenol, 597.00 μg/kg; and 3-methylphenol, 3529.00 μg/kg) [6,9]. The concentrations of 4-methylguaiacol (1395.64 μg/kg), 4-ethylguaiacol (534.46 μg/kg), and 3-methylphenol (96.84 μg/kg) were lower than the concentrations detected in mini-pig (4-methyl guaiacol, 1560.00 μg/kg; 4-ethylguaiacol, 1120.00 μg/kg; 3-methylphenol, 5140.00 μg/kg). Most of the phenolic compounds exhibited higher OAVs, which is consistent with previous studies [6,9,11].

In contrast to the compounds of a high concentration, 2,3,5-trimethylpyrazine (204.69 μg/kg), 2-methylpyrazine (142.89 μg/kg), methional (98.58 μg/kg), 3-methyl-2-cyclopenten-1-one (96.63 μg/kg), and octanal (76.77 μg/kg) were measured to have a lower concentration in THSL. Here, 2-acetyl-1-pyrroline (38.88 μg/kg) was first identified for the first time in smoke-cured pork leg. Though this aroma compound had a lower concentration in THSL, its low threshold value suggests its potential contribution to the overall aroma of THSL. Among the aroma-active compounds, the percentage of phenolic compounds (83.67%) was the highest, followed by aromatic compounds (10.02%), furan (2.15%), ketones (1.59%), aldehydes (1.14%), nitrogen compounds (1.14%), and sulfur compounds (0.29%). These results also suggest that phenolic compounds are the dominant aroma compounds in THSL.

To obtain deeper insight into the contribution of aroma-active compounds to the overall aroma profile of THSL, their OAVs (the ratio of concentration to odor threshold) were calculated. There were 27 odorants with OAVs ≥1 in THSL (Table 3), but 3 compounds (2-methylpyrazine, 2-acetylfuran, and 3-methyl-2-cyclopenten-1-one) had OAVs <1, indicating that they were insignificant to the overall aroma of THSL. These 24 odorants with OAVs in the range of 2–1472 were potent odorants for THSL aroma. (*E*)-2-Nonenal had the highest OAV (OAV = 1472) due to its significantly low odor threshold value (0.19 μg/kg), followed by 2-methoxy-4-vinylphenol (OAV = 542), guaiacol (OAV = 490), 2-acetyl-1-pyrroline (OAV = 324), 3-ethylphenol (OAV = 203), 2,6-dimethylphenol (OAV = 173), and (*E*)-2-octenal (OAV = 106). The results show that the overall aroma profiles of THSL consist of various aroma compounds with higher OAVs that provide more important contributions to the overall aroma.

### 3.4. Recombination Result

An aroma recombination experiment was conducted to validate the identification and quantification of the aroma-active compounds in THSL. All odorants with OAVs ≥1 were dissolved in an odorless capric triglyceride solution according to their measured concentrations. The sensory evaluation results of the recombination model and the THSL sample are shown in Figure 2a. A correlation analysis showed a 90.73% similarity between the recombination model and the THSL sample. However, there remained some differences between the milky, meaty, and smoky attributes. High significant difference (*p* < 0.01) in meaty attribute between the recombination model and THSL sample was observed. This might be due to the aroma compounds with meaty characteristic being digested or not being captured during SDE extraction.

### 3.5. Omission Test

To further corroborate the contributions of the potent odorants with OAVs ≥1 and to determine which compound are key odorants, 12 aroma omission models (M_1_–M_12_) were prepared. The results of the omission tests are shown in Table 4. A total of nine omission models were significantly different from the complete recombination models. In M_1_, the entire group of phenolic compounds was omitted due to their high FD factors and high OAVs. All panelists correctly detected the differences between the omitted model (M_1_) and the complete recombination model. The intensity of the smoky attribute was significantly (*p* < 0.001) decreased when the phenolic compounds were omitted, which indicates that phenolic compounds played an important role in the smoky notes of THSL. 2-Methoxy-4-vinylphenol, guaiacol, 3-ethylphenol, and 2,6-dimethylphenol, characterized by high FD factors and high OAVs, were also omitted in the four omission models of M_9_, M_10_, M_11_, and M_12_, respectively. The results showed that 14, 13, 13, and 12 panelists, respectively, were able to correctly detect the differences between the complete model and the omission models when these compounds were omitted (Table 4). However, they showed a different degree of contributions to the aroma profile of THSL due to their different properties. Over 13 out of 15 panelists detected the differences between the omitted model and the complete model when either 2-methoxy-4-vinylphenol or guaiacol were omitted. The intensities of the smoky and roasty attributes of the omitted models were also significantly decreased (*p* < 0.001). Therefore, 2-methoxy-4-vinylphenol and guaiacol were confirmed to be key odorants of THSL. In addition, significant differences (*p* < 0.01) between the omission models (M_11_ and M_12_) and the complete recombination model were observed, demonstrating that 3-ethylphenol and 2,6-dimethylphenol were also the key odorants of THSL, contributing to the smoky attribute. These results are consistent with those of previous studies, which showed that phenolic compounds play key roles in the smoky aroma of smoke-cured meat products [2,6,9,11]. Moreover, single phenolic compounds, including 2-methoxy-4-vinylphenol (M_9_), guaiacol (M_10_), 3-ethylphenol (M_11_), and 2,6-dimethylphenol (M_12_), were all confirmed to be key odorants of THSL. These conclusions are also in good agreement with the high OAVs and high FD factors determined for these compounds.

In M_2_, the entire group of ketone compounds was omitted due to their sweet and caramel-like characteristics. No significant difference was observed by the panelists between the complete recombination model and the omitted model of M_2_. This indicates that ketones are not the key odorants in THSL, which might be due to their lower OAVs. A similar result was observed when a group of aromatic compounds was omitted in M_4_, suggesting that aromatic compounds were also not key odorants in THSL. In M_3_, all of the aldehydes were omitted due to their high OAVs. In the results, 14 of 15 panelists could correctly detect the difference between the complete recombination model and the omitted model (M_3_). In addition, the intensity of the greasy attribute decreased significantly when aldehydes were omitted, suggesting that aldehydes contributed greatly to the greasy aroma of THSL. (*E*)-2-Nonenal (OAV = 1472) (M_6_) and octanal (OAV = 23) (M_7_) were further omitted to determine whether they play important roles in THSL aroma. In M_6_, a significant decrease (*p* < 0.001) in the intensity of greasy characteristics was observed by 14 of 15 panelists when (*E*)-2-nonenal was omitted, suggesting that (*E*)-2-nonenal is a key odorant in THSL. However, no significant difference was observed when octanal was omitted.

As previous studies have reported, 2-acetyl-1-pyrroline with a high FD factor is a key odorant in rice, bread, Jinhua ham, and smoked cooked lion [9,32,33,36]. Here, it was detected in THSL for the first time and had the fourth-highest OAV among all odorants in the THSL. A mixture of all the odorants, except for 2-acetyl-1-pyrroline, was produced for the M_5_. The triangle test showed that highly significant differences (*p* < 0.001) exist between the complete recombination sample and M_5_ and that 2-acetyl-1-pyrroline is a key odorant in THSL. In M_8_, the odorant with a high FD factor of 243 and a high intensity of cooked potato aroma was omitted. Twelve out of fifteen panelists correctly detected a distinction between the M_8_ and complete recombination models. A significant difference (*p* < 0.01) in the roasty attribute between the omitted model and the complete recombination model was observed, indicating that methional was the key odorant in THSL.

In summary, seven compounds, including methional, contributing to roasty notes; 3-ethylphenol, guaiacol, 2,6-dimethylphenol, and 2-methoxy-4-vinylphenol, contributing to smoky notes; (*E*)-2-nonenal, contributing to greasy notes; and 2-acetyl-1-pyrroline were confirmed as key odorants in THSL.

## 4. Conclusions

After salt-curing and smoke-curing, THSL had a more complex and diverse aroma profile, including meaty, woody, greasy, smoky, milky, spicy, and roasty notes. Through the application of GC–O/AEDA, 39 aroma-active compounds were detected, 30 of which were further quantified using standard curves. Twenty-seven odorants with OAV ≥1 were obtained. Among them, (*E*)-2-nonenal (OAV = 1,472), 2-methoxy-4-vinylphenol (OAV = 542), guaiacol (OAV = 490), 2-acetyl-1-pyrroline (OAV = 324), 3-ethylphenol (OAV = 203), and 2,6-dimethylphenol (OAV = 173) showed the highest OAVs. The recombination model had a 90.73% similarity with the original THSL sample, which validated the characterization of the aroma compounds in THSL. Omission experiments further confirmed that (*E*)-2-nonenal, 2-methoxy-4-vinylphenol, 3-ethylphenol, guaiacol, methional, 2-acetyl-1-pyrroline, and 2,6-dimethylphenol were key odorants in THSL. In addition, 2-acetyl-1-pyrroline was confirmed as the key odorant contributor of roasty in THSL for the first time.

## Figures and Tables

**Table 1:** The preference frequency results of the 10 traditional Hunan smoke-cured pork leg (THSL) samples with different smoke-curing times.

Samples	THSL-5	THSL-6	THSL-11	THSL-12	THSL-17	THSL-18	THSL-19	THSL-20	THSL-25	THSL-26
Frequency	0	0	0	3	8	14	10	7	2	1

**Table 2:** Aroma-active compounds identified in traditional Hunan Smoke-cured Pork Leg (THSL) by gas chromatography–olfactory-mass spectrometry (GC–O–MS).

No.	Compounds	RI		Identification	FD Factors	Odorant Descriptor
		TG-WAX	TG-5MS			
	Nitrogen compounds (3)					
N1	2-Methylpyrazine	1260	863	MS, RI, S, O	9	roasty, nutty
N2	2-Acetyl-1-pyrroline	1316	921	MS, RI, S, O	729	popcorn, grain, roasty
N3	2,3,5-Trimethylpyrazine	1385	1024	MS, RI, S, O	81	roasty, earthy
	Aldehydes (3)					
A1	Octanal	1291	1023	MS, RI, S, O	9	green, citrus
A2	(*E*)-2-Octenal	1393	1078	MS, RI, S, O	81	green leaf
A3	(*E*)-2-Nonenal	1589	1177	MS, RI, S, O	243	peanut, almond, fatty
	Ketones (6)					
K1	1-Octen-3-one	1315	-	MS, RI, S, O	3	mushroom
K2	3-Methyl-2-cyclopenten-1-one	1489	990	MS, RI, S, O	81	sweet, fruity
K3	3-Ethyl-2-cyclopenten-1-one	1677	-	MS, RI, S, O	81	caramel-like
K4	4,4-Dimethyl-2-cyclopenten-1-one	1768	964	MS, RI, O	27	caramel-like, bitter
K5	3-Methylacetophenone	1790	-	MS, RI, S, O	9	floral, sweet
K6	3-Ethyl-2-hydroxy-2-cyclopenten-1-one	1919	1136	MS, RI, S, O	81	sweet, caramel-like
	Sulfur compound (1)					
S1	Methional	1480	905	MS, RI, S, O	243	cooked potato
	Furan compounds (2)					
F1	2-Acetylfuran	1483	938	MS, RI, S, O	27	sweet, roast
F2	5-Methyl furfural	1593	986	MS, RI, S, O	27	green, sweet, grass
	Unknow compounds (3)					
U1	Unknown1	-	-	O	81	bitter, medicine
U2	Unknown2	-	-	O	27	cucumber, green,
U3	Unknown3	-	-	O	9	green
	Aromatic compounds (5)					
B1	1-Methylnaphthalene	1877	1300	MS, RI, S, O	81	medicinal, sweet, vanilla-like
B2	2-Methylnaphthalene	1908	1299	MS, RI, S, O	27	bitter
B3	2-Ethylnaphthalene	1962	1402	MS, RI, S, O	27	burning
B4	3,4-Dimethoxytoluene	1987	1281	MS, RI, O	9	green, dried grass
B5	3,4,5-Trimethoxytoluene	2069	-	MS, RI, S, O	243	bitter, earth, pungent
	Phenolic compounds (16)					
P1	Guaiacol	1888	1104	MS, RI, S, O	243	woody, sweet, smoky
P2	2-Methoxy-6-methylphenol	1897	1257	MS, RI, O	27	woody, sweet
P3	2,6-Dimethylphenol	1937	1122	MS, RI, S, O	81	smoky, burning
P4	4-Methyl guaiacol	1978	1208	MS, RI, S, O	729	sweet, wood, caramel-like, smoky
P5	2-Methylphenol	2028	1076	MS, RI, S, O	27	vanilla-like, woody
P6	4-Ethyl guaiacol	2048	1292	MS, RI, S, O	729	wood, smoky, caramel-like
P7	2,5-Dimethylphenol	2099	1168	MS, RI, S, O	243	butyric acid, stink, leather
P8	3,4-Dimethylphenol	2102	1194	MS, RI, S, O	2187	butyric acid, stinky, leathery
P9	3-Ethylphenol	2105	1158	MS, RI, S, O	6561	leathery, smoky
P10	2-Methoxy-4-propyl-phenol	2108	1382	MS, RI, S, O	729	green, cool, fresh
P11	3-Methylphenol	2109	1095	MS, RI, S, O	729	burning, leathery, stinky
P12	3,5-Dimethoxyphenol	2117	1189	MS, RI, S, O	243	rubbery, butyric acid
P13	2,3-Dimethoxyphenol	2128	-	MS, RI, S, O	243	rubbery, butyric acid
P14	2-Methoxy-4-vinylphenol	2208	1329	MS, RI, S, O	729	vanilla-like, smoky, woody
P15	2,6-Dimethoxyphenol	2283	1370	MS, RI, S, O	2187	leathery, green
P16	*trans*-2-Methoxy-4-propenyl-phenol	2367	1382	MS, RI, O	81	leathery, smoky

MS: identified by mass spectra; RI: calculated using a homologous series of *n*-alkanes (C_6_–C_30_); S: identified by standards; O: odor characteristics of the aroma compounds; “-”: not detected.

**Table 3:** Concentrations and odor activity values (OAVs) of the aroma-active compounds detected in traditional Hunan smoke-cured pork leg (THSL).

No.	Odorants	Concentration (μg/kg)	Threshold (μg/kg)	OAV
A1	(*E*)-2-Nonenal	279.75 ± 11.13	0.19 ^a^	1472
A2	(*E*)-2-Octenal	30.42 ± 1.54	4.00 ^b^	8
A3	Octanal	76.77 ± 22.61	3.40 ^a^	23
**Total**	Aldehydes	386.94		
P1	Guaiacol	1225.46 ± 72.44	2.50 ^a^	490
P3	2,6-Dimethylphenol	2455.15 ± 191.36	14.20 ^c^	173
P4	4-Methyl guaiacol	1395.64 ± 62.82	25.00 ^a^	56
P5	2-Methylphenol	2316.82 ± 248.34	45.00 ^a^	52
P6	4-Ethyl guaiacol	534.46 ± 41.54	16.00 ^a^	33
P7	2,5-Dimethylphenol	811.64 ± 55.08	400.00 ^c^	2
P8	3,4-Dimethylphenol	653.04 ± 59.91	17.00 ^c^	38
P9	3-Ethylphenol	344.81 ± 5.97	1.70 ^a^	203
P10	2-Methoxy-4-propyl-phenol	510.51 ± 80.98	157.00 ^c^	3
P11	3-Methylphenol	96.84 ± 14.66	15.00 ^a^	6
P12	3,5-Dimethoxyphenol	4368.41 ± 435.16	140.00 ^c^	31
P13	2,3-Dimethoxyphenol	1100.40 ± 30.37	170.00 ^c^	6
P14	2-Methoxy-4-vinylphenol	2761.99 ± 39.96	5.10 ^a^	542
P15	2,6-Dimethoxyphenol	9784.39 ± 852.69	263.00 ^a^	37
**Total**	Phenolic compounds	28,359.56		
N1	2-Methylpyrazine	142.89 ± 55.24	250.00 ^a^	<1
N2	2-Acetyl-1-pyrroline	38.88 ± 5.32	0.12 ^b^	324
N3	2,3,5-Trimethylpyrazine	204.69 ± 52.35	71.00 ^a^	3
**Total**	Nitrogen compounds	386.46		
B1	1-Methylnaphthalene	693.08 ± 48.08	10.75 ^c^	65
B2	2-Methylnaphthalene	206.49 ± 9.39	4.00 ^c^	52
B3	2-Ethylnaphthalene	222.57 ± 7.78	4.96 ^c^	55
B5	3,4,5-Trimethoxytoluene	2275.00 ± 29.69	120.00 ^c^	19
**Total**	Aromatic compounds	3397.14		
S1	Methional	98.58 ± 8.18	1.80 ^a^	55
**Total**	Sulfur compound	98.58		
K2	3-Methyl-2-cyclopenten-1-one	96.63 ± 52.23	300.00 ^b^	<1
K5	3-Methylacetophenone	236.84 ± 20.57	123.25 ^c^	2
K6	3-Ethyl-2-hydroxy-2-cyclopenten-1-one	205.09 ± 86.55	53.35 ^c^	4
**Total**	Ketone compounds	538.56		
F1	2-Acetylfuran	595.46 ± 35.50	1000.00 ^a^	<1
F2	5-Methyl furfural	133.84 ± 4.43	50.00 ^b^	3
**Total**	Furan compounds	729.30		

^a^ Odorant threshold values from Czerny, Christlbauer, Christlbauer, Fischer, Granvogl, Hernandez, and Schieberle (2008); ^b^ Odorant threshold values from (Burdock, 2010); ^c^ Odorant thresholds (in water) detected according to Czerny et al. (2008).

**Table 4:** Results of the omission tests.

No.	Aroma Profile Descriptions	Omitted Compounds	Correct Number in All	Significance
M_1_	Smoky, leathery	All of the phenolic compounds	15/15	***
M_2_	Caramel-like, sweet	All of the ketone compounds	7/15	-
M_3_	Green, grass, fatty	All of the aldehyde compounds	14/15	***
M_4_	Bitter, leathery	All of the aromatic compounds	8/15	-
M_5_	Popcorn, grain, roasty	2-acetyl-1-pyrroline	14/15	***
M_6_	Peanut, almond, fatty	(*E*)-2-nonenal	14/15	***
M_7_	green, citrus	octanal	10/15	-
M_8_	Cooked potato	methional	12/15	**
M_9_	Vanilla-like, smoky, woody	2-methoxy-4-vinylphenol	14/15	***
M_10_	Woody, sweet, smoky	guaiacol	13/15	***
M_11_	Leathery, smoky	3-ethylphenol	13/15	**
M_12_	Leathery, green	2,6-dimethylphenol	12/15	**

*** significance level *p* < 0.001; ** significance level *p* < 0.01; - no significant difference. The confidence interval was 95%.

## Supplementary Materials

The following are available online at https://www.mdpi.com/2304-8158/9/4/413/s1, Table S1: Standard curves of the aroma-active compounds.
