# Supplementary material for: Characterization of the Key Aroma Compounds in Traditional Hunan Smoke-Cured Pork Leg (Larou, THSL) by Aroma Extract Dilution Analysis (AEDA), Odor Activity Value (OAV), and Sensory Evaluation Experiments
Source: Foods. 2020 Apr 2;9(4):413. doi: 10.3390/foods9040413 (PMC7231236; doi:10.3390/foods9040413)
Supplement: Supplementary file 1 [file foods-09-00413-s001.pdf]

Table S1. Standard curves of the aroma-active compounds.

No.	Odorants	CAS	Quantitative ions	Standard Curves	R^2
N1	2-Methylpyrazine	109-08-0	94	$y = 4.0804x + 0.0018$	0.9978
N2	2-Acetyl-1-pyrroline	85213-22-5	111	$y = 0.0058x$	1.0000
N3	2,3,5-Trimethylpyrazine	14667-55-1	122	$y = 8.5197x + 0.0024$	0.9957
A1	(*E*)-2-Nonenal	18829-56-6	83	$y = 4.5829x + 0.0021$	0.9964
A2	(*E*)-2-Octenal	2548-87-0	55	$y = 6.9831x$-0.0044	0.9985
A3	Octanal	124-13-0	84	$y = 2.8516x + 0.0011$	0.998
K2	3-Methyl-2-cyclopenten-1-one	2758-18-1	96	$y = 0.7968x + 0.0021$	0.9968
K5	3-Methylacetophenone	585-74-0	119	$y = 1.9869x$-0.0126	0.9984
K6	3-Ethyl-2-hydroxy-2-cyclopenten-1-one	21835-01-8	126	$y = 2.2834x + 0.0037$	0.9942
S1	Methional	3268-49-3	104	$y = 40.546x + 0.0348$	0.9998
F1	2-Acetylfuran	1192-62-7	95	$y = 3.4342x$-0.0034	0.9994
F2	5-Methyl furfural	620-02-0	110	$y = 0.6537x + 0.0022$	0.9975
B1	1-Methylnaphthalene	90-12-0	142	$y = 0.7741x$-0.003	0.9988
B2	2-Methylnaphthalene	91-57-6	115	$y = 0.204x + 0.002$	0.9983
B3	2-Ethylnaphthalene	939-27-5	156	$y = 0.517x$-0.0084	0.9931
B5	3,4,5-Trimethoxytoluene	6443-69-2	182	$y = 3.2407x + 0.0078$	0.999
P1	Guaiacol	90-05-1	109	$y = 0.56x + 0.0027$	0.9969
P3	2,6-Dimethylphenol	576-26-1	107	$y = 4.9464x + 0.0024$	0.9989
P4	4-Methyl guaiacol	93-51-6	138	$y = 0.6177x + 0.0033$	0.9954
P5	2-Methylphenol	95-48-7	108	$y = 1.2178x + 0.0068$	0.9993
P6	4-Ethyl guaiacol	2785-89-9	152	$y = 0.2296x + 0.0031$	0.996
P7	2,5-Dimethylphenol	95-87-4	107	$y = 0.582x + 0.0022$	0.9973
P8	3,4-Dimethylphenol	95-65-8	122	$y = 29.356x$-0.0425	0.9987
P9	3-Ethylphenol	620-17-7	122	$y = 0.7462x + 0.0092$	0.9981
P10	2-Methoxy-4-propyl-phenol	2785-87-7	137	$y = 0.466x$-0.0041	0.9986
P11	3-Methylphenol	108-39-4	79	$y = 0.5271x + 0.0022$	0.9975
P12	3,5-Dimethoxyphenol	500-99-2	125	$y = 5.019x + 0.0023$	0.999
P13	2,3-Dimethoxyphenol	27257-08-5	196	$y = 4.3505x + 0.0034$	0.9999
P14	2-Methoxy-4-vinylphenol	7786-61-0	150	$y = 4.0954x + 0.0419$	0.9984
P15	2,6-Dimethoxyphenol	91-10-1	139	$y = 6.4053x + 0.0125$	0.9973

R^2, regression coefficient.
